# Interrater Reliability of Various Thyroid Imaging Reporting and Data System (TIRADS) Classifications for Differentiating Benign from Malignant Thyroid Nodules

**DOI:** 10.31557/APJCP.2019.20.4.1283

**Published:** 2019

**Authors:** Warinthorn Phuttharak, Arunnit Boonrod, Vivian Klungboonkrong, Thanatchaporn Witsawapaisan

**Affiliations:** *Department of Radiology, Faculty of Medicine, Khon Kaen University, Thailand. *

**Keywords:** Interrater reliability, agreement, thyroid nodule, TIRADS

## Abstract

**Background:**

Thyroid ultrasound(US) is used as the first diagnostic tool to assess the management of disease but is operator dependent. There have been few reports evaluating interrater variability in US assessment. Therefore, we evaluated interrater reliability in US assessment of thyroid nodules and estimated its diagnostic accuracy for various TIRADS systems.

**Methods:**

This retrospective study included 24 malignant nodules and 84 benign nodules from January 2015 to October 2017. Two blinded observers independently reviewed stored US images by using TIRADS. All analyses followed guidelines proposed by ACR-TR, Siriraj-TR and EU-TR systems. Interrater reliability was calculated using Cohen’s Kappa statistics. Diagnostic accuracy were also calculated.

**Results:**

Interobserver agreement showed substantial agreement for composition (K=0.616); echogenicity and echogenic foci showed fair agreement (K=0.327 and 0.288, respectively); margin showed slight agreement (K=0.143). Interrater reliability for the final assessment; moderate agreement for ACR-TIRADS system (K=0.500); fair agreement for EU-TIRADS system (K=0.209) and slight agreement (K=0.114) for Siriraj-TIRADS system. The diagnostic performance from the two observers; ACR-TIRADS system; sensitivities were 75% and 79.2%, specificities were 58.3% and 56%, positive predictive value (PPV) were 34% and 33.9% and negative predictive value (NPV) were 89.1% and 90.4%. For the Siriraj-TIRADS system, sensitivities were 41.7% and 25%, specificities were 84.5% and 89.3%, positive predictive value (PPV) were 43.5% and 40% and negative predictive value (NPV) were 83.5% and 80.6%. For the EU-TIRADS system, sensitivities were 45.8% and 66.7%, specificities were 79.8% and 72.6%, positive predictive value (PPV) were 39.3% and 41% and negative predictive value (NPV) were 83.8% and 88.4%.

**Conclusion:**

The ACR-TIRADS had highest interobserver agreement, a trend to have highest sensitivity and negative predictive value for diagnosis of malignant thyroid nodules. Siriraj-TIRADS had higher specificity and accuracy, but lower interobserver agreement.

## Introduction

Thyroid nodules are an increasingly common finding during imaging examination of the neck (Grani et al., 2018). The estimated prevalence by palpation alone ranges from 4% to 7%, whereas ultrasonography (US) detects nodules in 20% to 76% of the adult population, particularly with the current use of high-resolution US techniques (Popoveniuc et al., 2012). Nodules can be benign or malignant. Some studies have shown that less than 10% of thyroid nodules are malignant (Boniface et al., 2013). Fine-needle aspiration (FNA) biopsy plays a major role in the differential diagnosis, but its execution needs to be selective, due to associated cost, potential non-diagnostic results and the risk of overdiagnosis (Grani et al., 2018).

A number of classification systems have been developed to estimate the likelihood of malignancy but ultrasonography is a relatively subjective diagnostic method, and observers may have different opinions when they describe and interpret lesions (Park et al., 2009). There are only a small number of reports evaluating observer variability in US assessment (Choi et al., 2010).

Therefore, we conducted a cross-sectional retrospective analysis of recorded US images to evaluate interrater reliability between two radiologists using single US features, for final assessment and diagnostic performance among three TIRADS classification systems.

## Materials and Methods

*Study population*

The study protocol was approved by the Human Ethics Research Committee, Khon Kaen University (HE 601501) and did not require patient approval or informed consent for the review of patient images. All patients had earlier given written informed consent for the US-guided FNAB. It was a cross-sectional retrospective study carried out at Srinagarind Hospital (Thailand) from January 2015 to October 2017. During this period, US-guided FNAB was performed in 157 thyroid nodules in 139 patients. Patients were included if they (a) underwent US-guided FNA and had benign or malignant results at cytologic examination; but for benign cytology group follow up US examination after at least 12 months without a significant change, such as new detection of suspicious US features or significant increase in nodular size (50% or more increase in nodular volume) (b) was performed US of neck before US-guided FNAB within 3 months period. Among the 157 nodules examined, 49 were excluded because they were suspicious for malignancy (n=2), atypia of undetermined significance or follicular lesion of undetermined significance (n=10), follicular neoplasm or suspicious for a follicular neoplasm (n=2), non diagnostic or unsatisfactory (n=35) at cytologic examination but did not undergo surgery. The 24 malignant nodules included 20 cases of papillary carcinoma, 2 cases of Hurthle cell carcinoma and 2 cases of follicular carcinoma.

*Imaging and Imaging analyses*

Ultrasound of neck was obtained using 9-12 MHz linear-array transducer (GE healthcare LOGIQ 9 and Hitachi ALOKA Prosound F75). Conventional imaging was performed in all patients. Real time ultrasound was performed by heterogeneously experienced radiologists. 

Two radiologists independently reviewed the ultrasonography and were blinded to the number of benign and malignant lesions as well as to the clinical findings and the cytopathologic results. The readers had 2 years and more than 10 years experience examining sonograms of thyroid glands in a standard manner.

For gray-scale US, the nodules were evaluated for composition, echogenicity, shape, size, margin and echogenic foci.

- Composition of the nodule was categorized according to the ratio of cystic portion to the solid portion in the nodule and was classified as “solid” (composed entirely or nearly entirely of soft tissue, with only a few tiny cystic spaces), “predominately solid” (composed of soft tissue components occupying 50% or more of the volume of the nodule), “predominately cystic” (composed of soft tissue components occupying less than 50%), “cystic” (entirely fluid filled) or “mixed cystic and solid”. Spongiform was defined as predominately of tiny cystic spaces more than 50%.

- Echogenicity of the nodule was assessed by comparing echogenicity of the nodule with that of thyroid parenchyma and strap muscle, and was classified as “hyperechoic” (increased echogenicity relative to thyroid tissue), “isoechoic” (similar echogenicity relative to thyroid tissue), “hypoechoic” (decreased echogenicity relative to thyroid tissue) or “very hypoechoic” (decreased echogenicity relative to adjacent neck musculature)

- Shape of the nodule was categorized as “taller-than-wide” (ratio of >1 in the anteroposterior diameter to the horizontal diameter when measured in the transverse plane) or “wider-than-tall” (anteroposterior diameter of nodule equal to or less than its horizontal diameter on transverse plane).

- Size of the nodule is assessed by use maximal diameter on the axial plane in centimeters.

- Margins of the nodule was categorized as “smooth” (uninterrupted, well-defined, curvilinear), “irregular margin” (the outer border of the nodule is spiculated, jagged, or with sharp angles with or without clear soft tissue protrusions into the parenchyma), “lobulated” (border has focal rounded soft tissue protrusions that extend into the adjacent parenchyma), “ill-defined” (border of the nodule is difficult to distinguish from thyroid parenchyma), “halo” (border consists of a dark rim around the periphery of the nodule), “extrathyroidal extension” (nodule extends through the thyroid capsule).

- Echogenic foci within the nodule was assessed regarding its size and was classified as “punctate echogenic foci” (“Dot-like” foci having no posterior acoustic posterior artifacts, 1 mm or less), “macrocalcifications” (large enough to result in posterior acoustic shadowing), “peripheral calcifications” (occupy the periphery of the nodule), “comet-tail artifacts” (type of reverberation artifact that deeper echoes become attenuated and are displayed as decreased width, resulting in a triangular shape).

Two parameters were not rated by either of the readers: (1) nodule diameters, which had been recorded during the original scan and were visible on the stored images (2) nodule shape, wider-than-tall or taller-than-wide, the classification of which is strictly dependent on the nodule dimensions. These features were excluded from analyses of interobserver agreement.

For each nodule, the ratings of each readers (together with those recorded during the original examination for nodule size and shape) were used in an algorithm to classify the nodule according to the following three systems: the TIRADS system developed by the American College of Radiologists (ACR) (Tessler et al., 2017); Siriraj-TIRADS system (Dittapong et al., 2017) and the EU-TIRADS system proposed by the European Thyroid Association (Russ et al., 2017).

**Table 1:** Interobserver Agreement for Single US Features

US characteristic	K-value	P-value
Composition	0.616 (0.521;0.664)	<0.05
Echogenicity	0.327 (0.246;0.479)	<0.05
Margin	0.143 (0.039;0.284)	<0.05
Echogenic foci	0.288 (0.218;0.379)	<0.05

**Figure 1:**
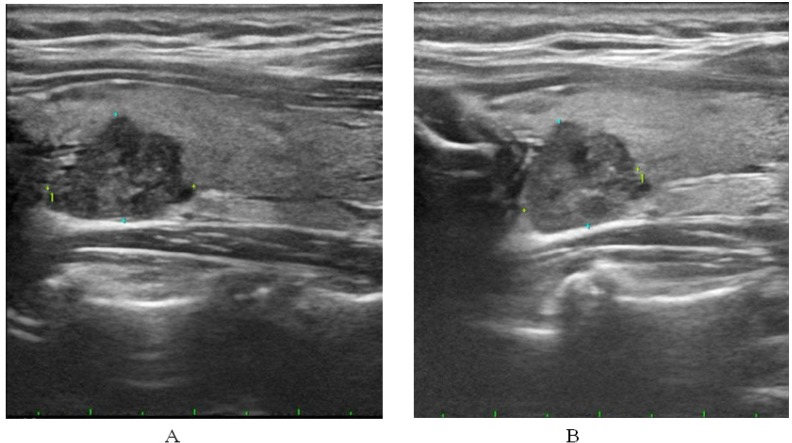
Papillary Carcinoma in 57-Year-Old Woman. Axial (A) and sagittal (B) images representing a case that showed near perfect interobserver agreement for US features, described as having solid, marked hypoechogenicity, irregular margin and having microcalcification by two observers

**Figure 2:**
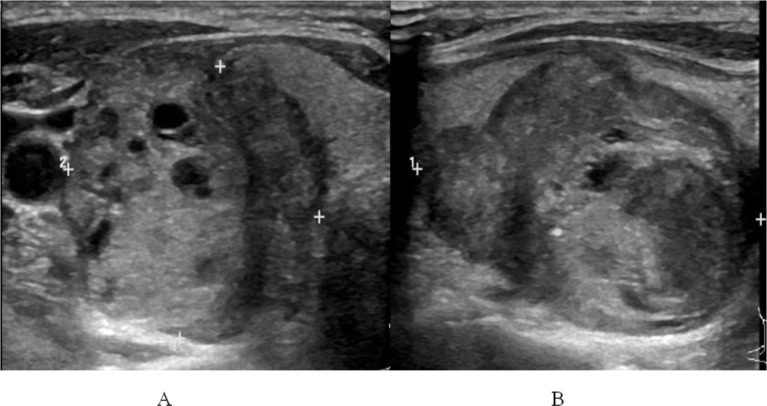
Papillary Carcinoma in 56-Year-Old Woman. Axial (A) and sagittal (B) images representing a case that showed poor interobserver agreement for margin. The first observer described this case as having lobulated/irregular margin and the other described as smooth margin

**Figure 3:**
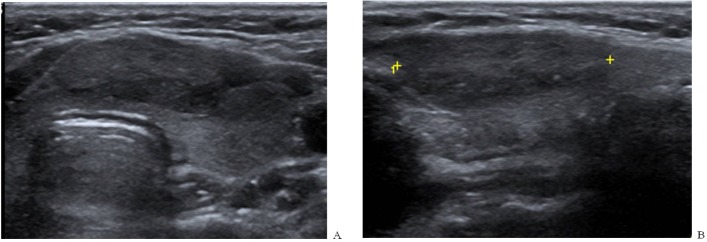
Benign Thyroid Nodule in 52-Year-Old Woman. Axial (A) and sagittal (B) images representing case that showed poor interobserver agreement for echogenic foci. The first observer described this case as having punctate echogenic foci and the other described as having no echogenic foci

**Table 2:** Interobserver Agreement between Observers for Each Category of Various TIRADS Systems

TIRADS	ACR- TR	P-value	Siriraj-TR	P-value	EU-TR	P-value
TIRADS 1	0.852	< 0.05	0.652	< 0.05	N/A	N/A
TIRADS 2	0.525	< 0.05	0.14	0.073	0.274	< 0.05
TIRADS 3	0.375	< 0.05	-0.031	0.627	0.167	< 0.05
TIRADS 4	0.229	< 0.05	0.106	0.136	0.092	0.168
TIRADS 5	0.177	< 0.05	N/A	N/A	0.2	< 0.05

**Table 3:** Interobserver Agreement for Final Assessment of TIRADS Classification Systems for the Diagnosis of High Suspicious Thyroid Nodules

TIRADS	K-value	P-value
ACR-TIRADS 4-5	0.500 (0.366;0.623)	<0.05
Siriraj-TIRADS 4-5	0.114 (0.024;0.285)	0.11
EU-TIRADS 5	0.209 (0.126;0.318)	<0.05

**Table 4:** Diagnostic Performance for the Diagnosis of Malignant Thyroid Nodules of Each TIRADS Classification Systems

	Observer 1			Observer 2		
	ACR-TR	Siriraj-TR	EU-TR	ACR-TR	Siriraj-TR	EU-TR
True positive	18	10	11	19	6	16
False positive	6	14	13	5	18	8
True negative	49	71	67	47	75	61
False negative	35	13	17	37	9	23
Sensitivity (%)	75 (53.3;90.2)	41.7 (22.1;63.4)	45.8 (25.6;67.2)	79.2 (57.8;92.9)	25 (9.77;46.7)	66.7 (44.7;84.4)
Specificity (%)	58.3 (47.1;69.0)	84.5 (75;91.5)	79.8 (69.6;87.7)	56 (44.7;66.8)	89.3 (80.6;95)	72.6 (61.8;81.8)
PPV (%)	34 (21.5;48.3)	43.5 (23.2;65.5)	39.3 (21.5;59.4)	33.9 (21.8;47.8)	40 (16.3;67.7)	41 (25.6;57.9)
NPV (%)	89.1 (77.8;95.9)	83.5 (73.9;90.7)	83.8 (73.8;91.1)	90.4 (79.0;96.8)	80.6 (71.1;88.1)	88.4 (78.4;94.9)
Accuracy (%)	62.04 (52.2;71.2)	75.0 (65.8;82.8)	72.2 (62.8;80.4)	61.1 (51.3;70.3)	75.0 (65.8;82.8)	71.3 (61.8;79.6)

*Ultrasound guided FNAB*

After US evaluation of the thyroid gland, US-guided FNAB was performed within 3 months period. At our institution, US-guided FNAB is performed in either the thyroid nodule with suspicious US features or the largest thyroid nodule if no suspicious US features are detected. US-guided FNAB was performed with 24-gauge needle attached to a 10-ml disposable plastic syringe. Each lesion was aspirated at least one attempt. Materials obtained from aspiration biopsy were expelled onto six glass slides and smeared. Four slide smears were placed immediately in 95% alcohol for Papanicolaou staining, 2 slides were dry fixed and sent to the pathology laboratory. Cytopathologists were not on site during the biopsy. During the study period, the cytology reports at our institution were classified as (a) benign follicular nodule (b) malignancy (c) suspicious for malignancy (d) atypia of undetermined significance or follicular lesion of undetermined significance (e) follicular neoplasm or suspicious for a follicular neoplasm (f) non diagnostic or unsatisfactory according to Bethesda system 2009. Histology was performed if cytology was indeterminate or suggestive of malignancy.

*Data and statistical analysis*

We assessed interrater reliability at the level of single features of the nodule and final assessment of TIRADS based on each of the three classification systems. Interobserver agreement was evaluated using Cohen’s kappa statistic. Values less than 0.20 are considered indicative of slight agreement; 0.21-0.40, fair agreement; 0.41-0.60, moderate agreement; 0.61-0.80, substantial agreement and 0.81-1.00, near-perfect agreement. 

For the assessment of diagnostic performance of the guidelines, sensitivity, specificity, positive predictive value and negative predictive value were calculated using cyto-histology as the reference standard. For all statistics, 95% confidence intervals (CI) were also calculated. A P-value less than 0.05 was considered to indicated a significant difference. All analyses were performed with Stata version 10.

## Results

This study included 108 thyroid nodules in 94 patients (7 men; 7.45% and 87 women; 92.55%). Of the 94 patients, 81 patients had one thyroid nodule, 12 had two nodules and 1 had three nodules. The mean age group was 51.6 years (mean±SD 51.6±13.08, min 10 years, max 75 years). The mean size of the nodules was 2.12 cm (mean±SD 2.12±1.2, min 0.46 cm, max 8.0 cm).

A summary of interobserver agreement for the single US features is shown in Table 1. There was substantial agreement for composition (K=0.616) and there was fair agreement for echogenicity and echogenic foci (K=0.327 and 0.288, respectively). The margin showed slight agreement (K=0.143).

Summary of interobserver agreement between observers for each category of various TIRADS systems is shown in Table 2. For ACR-TR; TIRADS 1 shows near-perfect agreement (K=0.852), TIRADS 2 shows moderate agreement (K=0.525), TIRADS 3 and 4 show fair agreement (K=0.375 and 0.229, respectively) and TIRADS 5 shows slight agreement (K=0.177). For Siriraj-TR; TIRADS 1 shows substantial agreement (K=0.650), TIRADS 2 and 4 show slight agreement without statistical significance (K=0.140 and 0.106, respectively) and there was no agreement for TIRADS 3 but no statistical significance (K=-0.031). For EU-TR; there were fair agreement for TIRADS 2 (K=0.274) and slight agreement for TIRADS 3, 4 and 5 (K=0.167, 0.092 and 0.200, respectively).

The summary of interobserver agreement for the final assessment of TIRADS classification systems for the diagnosis of high suspicious nodules is shown in Table 3. There was moderate agreement for ACR-TIRADS system (K=0.500) and there was fair agreement for EU-TIRADS system (K=0.209). The Siriraj-TIRADS system showed slight agreement (K=0.114) without statistical significance (P-value=0.11).

The diagnostic performance from the two observers are summarized in Table 4. For the ACR-TIRADS system, sensitivities were 75% and 79.2%, specificities were 58.3% and 56%, positive predictive value (PPV) were 34% and 33.9% and negative predictive value (NPV) were 89.1% and 90.4%. For the Siriraj-TIRADS system, sensitivities were 41.7% and 25%, specificities were 84.5% and 89.3%, positive predictive value (PPV) were 43.5% and 40% and negative predictive value (NPV) were 83.5% and 80.6%. For the EU-TIRADS system, sensitivities were 45.8% and 66.7%, specificities were 79.8% and 72.6%, positive predictive value (PPV) were 39.3% and 41% and negative predictive value (NPV) were 83.8% and 88.4%.

## Discussion

Several classification systems have been developed to help physicians and radiologists in differentiate between benign and malignant thyroid nodules which may need further US-guided FNAB. However, ultrasound is relatively operator dependent to accurately describe key nodule features. There have been small number of reports evaluating observer reliability in US assessment (Choi et al., 2010). The aim of this study was evaluating interrater reliability for two radiologists in three TIRADS classification systems; ACR-TIRADS, Siriraj-TIRADS and EU-TIRADS system. 

Based on US findings of gray-scale technique, each nodule was classified as being malignant or benign according to established criteria. Nodules displaying marked hypoechogenicity (Hong et al., 2009; Kwak et al., 2011; Boniface et al., 2013; Ricardo et al., 2017), solid appearance (Kwak et al., 2011; Ricardo et al., 2017), microcalcification (Kwak et al., 2011; Boniface et al., 2013; Ricardo et al., 2017), taller-than-wide (Hong et al., 2009; Kwak et al., 2011; Boniface et al., 2013), irregular/lobulated margin (Kwak et al., 2011; Boniface et al., 2013; Ricardo et al., 2017) were considered malignant. Nodules with spongiform appearance, well-defined margin or macrocalcification were classified as benign. 

In the current study, the interobserver reliability for two radiologists showed a highest agreement for the composition (substantial), followed by echogenicity and echogenic foci (moderate), but showed lowest agreement for margin (slight).

Similar to the study by Park et al., (2012), three radiologists showed near-perfect observer variability for the composition, moderate agreement for echogenicity and echogenic foci. The margin showed lowest agreement. In the study of Park et al., (2009), five observers showed substantial agreement for composition, moderate agreement for echogenicity and echogenic foci and poor agreement for margin. According to a study by Kim et al., (2010), the interobserver agreement among five faculties showed substantial agreement for composition, moderate agreement for echogenicity and calcification and fair agreement for margin. The interobserver agreement among four residents were poorer except for calcification where moderate agreement was achieved.

This contrasts with Grani et al., (2018) who found interobserver reliability between two clinicians had substantial agreement for calcification. In addition, there was moderate agreement for echogenicity, composition and margin. Furthermore, Grani et al., (2018) performed two sessions of analysis before the study and after completion of the training session. However, the nodules included in the study were all benign.

In the study of Choi et al., (2010), four radiologists showed moderate agreement for composition, margin and calcification with a fair agreement for echogenicity.

In present study, the final assessment of TIRADS classification systems in detecting high suspicious nodule showed moderate agreement for ACR-TIRADS system, fair agreement for EU-TIRADS system and slight agreement for Siriraj-TIRADS system. Difference from the study by Grani et al., (2018) which described substantial agreement for EU-TIRADS system which is highest among five TIRADS classification systems and ACR-TIRADS system showed moderate agreement. There has been no study in interobserver agreement for Siriraj-TIRADS system, which was recently proposed by Songsaeng et al., (2017). The differences in the system structures may explain the higher interobserver agreement for each category of ACR-TIRADS classification system which contained more details then observers can apply to all nodules, compared to EU-TIRADS and Siriraj-TIRADS system.

Regarding diagnostic performance of each TIRADS in this study, Sensitivity of ACR-TIRADS seemed to be higher than Siriraj-TIRADS and EU-TIRADS. However, due to 95% confidence intervals overlapping ACR-TIRADS has a trend towards being more sensitive than the others. For specificity, Siriraj-TIRADS had higher specificity than ACR-TIRADS without overlapping 95% confidence intervals. But when compared to EU-TIRADS, specificity of Siriraj-TIRADs seemed to be higher with overlapping 95% confidence intervals. This may suggest Siriraj-TIRADS has a trend towards being most specific. For accuracy, Siriraj-TIRADS seemed to be highest but due to overlapping of 95% confidence intervals Siriraj-TIRADS has a trend towards being more accuracy than the other classifications. 

There were several limitations of the current study. First of all, this is a retrospective study of patients who underwent ultrasound-guided FNAB and selective bias may have existed in recruiting patients to include in the study because all nodules were suspicious and submitted to US-guided FNAB. Second, the observers did not perform or evaluate real-time ultrasound themselves and only interpreted the static images. Thus, the observers could not take advantage of certain real-time ultrasound features. Third, intraobserver variability in different time point were not assessed. Other limitations are that the high percentage (22.22%) of carcinoma in the present study, might have resulted in a bias and 12 month US follow-up intervals might be too short to confirm the benignity of the thyroid nodules in the benign cytology group. Furthermore, this study does not provide data about increasing used ancillary techniques, like color doppler and elastosonography.

In summary, ACR-TIRADS had higher interobserver agreement, a trend to have highest sensitivity and negative predictive value for diagnosis of malignant thyroid nodules. Siriraj-TIRADS had a trend to higher specificity and accuracy, but lower interobserver agreement.

## Disclosure

No conflict of interest.
